# CRISPR/Cas9-Mediated *pds* Knockout in Potato Reveals Network-Level Transcriptomic Reorganization Beyond Pigment Loss

**DOI:** 10.3390/plants15010096

**Published:** 2025-12-28

**Authors:** Xianjun Lai, Yuxin Xiang, Siqi Liu, Yandan Zhang, Yizheng Zhang, Zihan Chen, Shifeng Liu, Lang Yan

**Affiliations:** 1 Panxi Crop Improvement Key Laboratory of Sichuan Province, College of Agriculture Science, Xichang University, Liangshan 615000, China; 2 Sichuan Key Laboratory of Molecular Biology and Biotechnology, College of Life Sciences, Sichuan University, Chengdu 610065, China

**Keywords:** CRISPR/Cas9, *pds* gene knockout, potato (*Solanum tuberosum*), albino phenotype, transcriptomic reprogramming

## Abstract

Background: The phytoene desaturase gene is a classical visual marker for validating CRISPR/Cas9 genome editing in plants, as its loss of function produces a readily scorable albino phenotype. While the biochemical basis of pigment loss is well established, it remains unclear whether *pds* knockout elicits transcriptomic changes extending beyond carotenoid biosynthesis. Resolving this question is essential for correctly interpreting *pds*-based editing outcomes and for assessing the robustness of phenotype-only screening approaches. Methods: A CRISPR/Cas9 editing platform targeting *pds* was established in diploid potato. Albino, non-albino edited, and wild-type tissues were subjected to RNA-seq profiling. Differential expression, functional enrichment, and weighted gene co-expression network analysis were integrated to resolve phenotype-associated transcriptional modules, and hierarchical regulatory layers underlying albinism. Results: CRISPR/Cas9-mediated disruption of *pds* in potato-generated stable albino phenotypes and revealed extensive transcriptomic reprogramming that was not limited to pigment loss. Albino tissues exhibited more than 9700 differentially expressed genes relative to both wild-type and non-albino edited tissues, whereas non-albino edits showed substantially fewer changes. Functional enrichment demonstrated pervasive suppression of photosynthesis and carbon metabolism alongside activation of secondary metabolism, stress responses, hormone signaling, and cell wall remodeling. WGCNA and cross-validation resolved these changes into distinct, phenotype-associated regulatory layers: MEorangered4 captured coordinated repression of starch and sucrose metabolism (*r* = −0.998), MEdarkgreen marked albino-linked activation of secondary metabolism and barrier biogenesis (*r* = 0.855; overlap with Albino Core set, OR = 23.65), while MEblack and MEgrey60 reflected downregulation of stress signaling, proteostasis, and hormone-integrative control and were enriched in transgenic–background-associated gene sets. Conclusions: *pds* knockout in potato is accompanied by broad transcriptomic changes beyond pigment biosynthesis, suggesting that albinism involves coordinated regulatory and metabolic adjustment under plastid dysfunction rather than pigment loss alone. These results refine the use of *pds* as a visual editing marker and provide a framework for linking localized genome edits to coordinated network-level transcriptional responses in plants.

## 1. Introduction

Potato (*Solanum tuberosum* L.) ranks as the fourth-most important food crop worldwide after wheat, rice, and maize (FAOSTAT, 2024) [1]. Its high caloric yield per unit area, adaptability to diverse environments, and diverse applications make it central to global food security [2,3]. In recent years, diploid hybrid inbred lines and true seed propagation have accelerated genetic improvement, aiming to overcome the genetic complexity and slow progress associated with tetraploidy and vegetative propagation [4,5]. However, the development of high-throughput and genotype-flexible gene editing pipelines remains a key bottleneck [6,7]. In particular, the strong genotype dependence of transformation and regeneration, combined with variable editing efficiency, hampers the routine deployment of CRISPR-based tools across germplasm.

A common strategy to assess editing efficiency involves targeting a marker gene whose loss-of-function produces a visible phenotype. The phytoene desaturase (*pds*) gene, encoding a key enzyme in carotenoid biosynthesis, is widely used for this purpose. Its knockout disrupts carotenoid and chlorophyll synthesis, resulting in albino tissues that serve as intuitive visual indicators of editing activity [8,9]. Numerous studies on crops, such as tomato [10], rice [11], and banana [12], have employed *pds* as a standard target to evaluate editing efficiency and optimize transformation conditions. In potato, *pds* editing has also yielded albino mutants in multiple cultivars, helping validate regeneration protocols and editing pipelines [9,13].

However, despite widespread use of *pds* as a test locus, most existing studies focus on editing efficiency or morphological outcomes, with limited attention paid to the transcriptional consequences and the underlying molecular mechanisms of albinism. Importantly, plastid-to-nucleus communication (also known as retrograde signaling) plays a central role in synchronizing nuclear gene expression with plastid development and function, both under normal growth and stress conditions. Various plastid-derived signals, including reactive oxygen species (ROS), tetrapyrrole intermediates, and carotenoid-derived apocarotenoids, modulate nuclear transcriptional programs involved in photosynthesis, hormone signaling, and metabolic homeostasis [14,15,16]. Disruption of plastid function, such as that caused by loss of *pds*, can impair apocarotenoid biosynthesis and retrograde signaling, thereby potentially altering nuclear transcriptional regulation beyond pigment-related pathways. This mechanistic framework raises important questions: Does the albino phenotype solely reflect pigment loss, or does it involve broader disruptions to plastid integrity, energy metabolism, and nuclear regulation? What molecular differences distinguish albino from non-albino mutants with similar genotypes? Do green or mosaic-edited lines activate compensatory responses that buffer the phenotypic outcome?

To address these questions, we designed a study that combines genome editing, phenotypic characterization, and transcriptomic dissection of potato *pds* mutants. Specifically, we (i) constructed and optimized a high-efficiency CRISPR/Cas9 editing pipeline in potato using *pds* as a visual marker for system validation; (ii) generated and classified edited lines into albino and non-albino groups based on phenotypic outcomes; and (iii) performed RNA-seq and network analysis to identify core transcriptional modules, differentially expressed pathways, and putative buffering responses. By moving beyond phenotypic scoring to a network-informed transcriptomic analysis, our study shows that *pds* knockout affects not only carotenoid and chlorophyll biosynthesis but is also associated with coordinated transcriptional changes in the photosynthetic apparatus, plastid-to-nucleus signaling, protein synthesis, and oxidative homeostasis. Our findings suggested that the albino phenotype represents a visible outcome of multilayered regulatory perturbation rather than pigment loss alone, offering new mechanistic insights and extending the functional landscape of *pds* in plant systems biology and genome editing.

## 2. Results

### 2.1. Establishment of a Visual CRISPR/Cas9 Editing System Targeting pds in Potato

**Figure 1:**
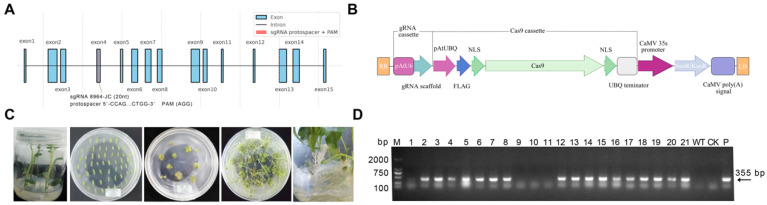
Construction of the CRISPR/Cas9 vector targeting *pds*, transformation workflow, and molecular verification in potato. (**A**) Schematic representation of the *pds* target site, with the sgRNA located in exon 4. The protospacer, PAM motif, and predicted Cas9 cleavage site were indicated. (**B**) Map of the binary vector pCAMBIA2300-CAS9-8964. (**C**) Workflow of Agrobacterium-mediated transformation in potato. (**D**) Representative diagnostic PCR of T-DNA insertion using primers 8964-F/8964-R. WT, no-template control (NTC), and plasmid-positive control (“+”) were shown. M indicates the DNA marker.

To establish a high-efficiency and visually scorable CRISPR/Cas9 editing platform in potato, we selected the *pds* gene as the target and constructed a binary expression vector, pCAMBIA2300-CAS9-8964, carrying a single-guide RNA (sgRNA) cassette. The sgRNA was designed to target exon 4 of the *pds* gene, with the Cas9 cleavage site positioned 3 bp upstream of the PAM sequence, as illustrated in Figure 1A. The vector architecture (Figure 1B) included an AtU6 promoter driving the sgRNA scaffold and an *Arabidopsis* ubiquitin (UBQ) promoter driving a FLAG-tagged Cas9 coding sequence containing dual nuclear localization signals (NLSs). For selection, a neomycin phosphotransferase II (*NPTII*) gene conferring kanamycin resistance was placed under the control of a CaMV 35S enhancer-promoter and CaMV polyadenylation signal. After assembly, the sgRNA cassette and key junctions were validated by Sanger sequencing, confirming the expected band sizes and sequence accuracy (Supplementary Figure S1 and Table S1). To assess potential off-target risk of the designed sgRNA, in silico off-target prediction (≤ 3 mismatches, cutting frequency determination > 0.2, localization within annotated exon regions) identified 25 potential off-target loci (Supplementary Table S2). These sites were subsequently amplified and examined by Sanger sequencing, and no mutations were detected at any of these loci.

Agrobacterium-mediated transformation was performed using the diploid potato genotype DM as the recipient (Figure 1C). Following a 3-day co-cultivation, explants were subjected to selection on medium containing 100 mg/L kanamycin and 300 mg/L Timentin, yielding 21 regenerated shoots. T-DNA diagnostic PCR using primers 8964-F/8964-R identified 17 transgenic lines (Figure 1D), corresponding to a transformation efficiency of 80.95%. Among these, 15 lines successfully rooted and were advanced to phenotypic assessment and molecular validation.

### 2.2. On-Target Genotyping and Albino Phenotype

**Figure 2:**
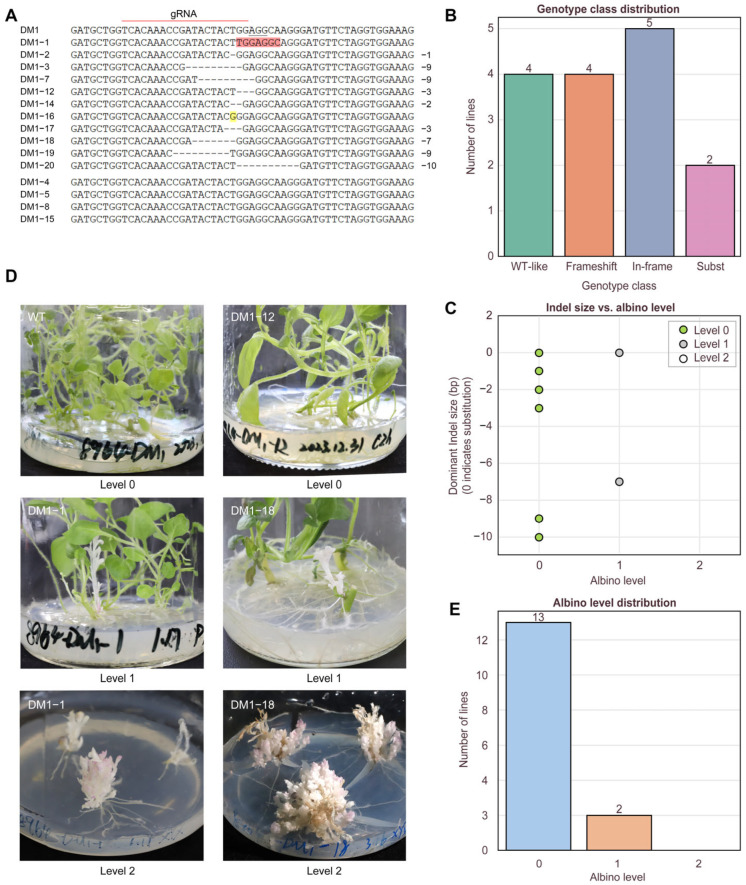
On-target genotyping and albino phenotype across edited lines. (**A**) Representative Sanger alignments at the *pds* target (WT vs. edited), with protospacer, PAM, and cut site indicated; mixed peaks denote mosaic edits; pink and yellow indicate base substitutions. (**B**) Genotype class distribution. (**C**) Spectrum of deletion sizes (−1/−2/−3/−7/−9/−10 bp) based on dominant Sanger-called alleles, The white circle (Level 2) is not shown in (**C**) but is included in the legend to indicate three levels. (**D**) Representative images of phenotypic classes defined by albino level scoring (0–2), including the DM1-1 and DM1-18 examples showing co-occurrence of albino and non-albino tissues on the same plant. The last two images show fully albino plantlets obtained from albino branches of chimeric lines through subculture, supporting stable inheritance of the albino phenotype. (**E**) Distribution of AlbinoLevel across lines.

To characterize the editing efficiency and mutation spectrum at the target site, all positive transgenic lines were subjected to PCR amplification using primers PDStec1-F and PDStec1-R, followed by Sanger sequencing and TIDE deconvolution. Sequence alignment was performed within a ±30–50 bp window flanking the predicted Cas9 cleavage site to assess editing events in all 15 lines. Representative chromatograms revealed overlapping peaks downstream of the cut site, indicative of mosaic editing or the presence of multiple alleles within individual lines (Supplementary Figure S2). Based on TIDE deconvolution, manual inspection, and sequence comparisons with the wild-type *pds* locus identified editing events in 11 of the 15 PCR-positive lines, while the remaining four lines showed sequences identical to the wild type (Figure 2A). Among the 11 edited lines, 9 carried insertion/deletion (indel) mutations, with frameshift mutations accounting for 4 out of 9 (44.4%) of these cases. These frameshift variants were predicted to cause premature termination and loss of protein function. The remaining five indel lines exhibited in-frame mutations (55.6%), potentially resulting in hypomorphic or partially functional alleles. In addition, two lines carried nucleotide substitutions (Figure 2B). Analysis of the indel types revealed that the majority of deletions were short (1–10 bp), encompassing seven distinct alleles: −1 bp, −2 bp, −3 bp, −7 bp, −9 bp, and −10 bp (Figure 2C).

To evaluate the phenotypic consequences of CRISPR/Cas9-mediated *pds* editing, we monitored transgenic lines over two successive subcultures under identical conditions. Phenotypes were scored based on albino severity using a three-level albino index: AlbinoLevel = 0 (fully green), 1 (partial albino branches), or 2 (fully albino plantlets). Among the 11 edited lines, two (DM1-1, DM1-18) exhibited clearly albino lateral branches (AlbinoLevel = 1), while the main stems remained green. Notably, DM1-18 harbored a frameshift mutation and showed partial albinism, while DM1-1, which carried both substitution (6 bp) and low-frequency deletions (−4 and −7 bp), also exhibited chimeric albino branches (AlbinoLevel = 1). These two chimeric individuals provided an internal phenotypic contrast within a single plant (Figure 2D). The albino branches displayed a dwarf phenotype characterized by highly compact growth, excessive branching, extremely small leaves, and characteristic pink pigmentation along the leaf margins. Upon excision and re-propagation, these albino branches gave rise to similarly dwarfed plantlets lacking a dominant main stem and forming entirely white microtubers, suggesting stable maintenance of the edited phenotype across subculture cycles. The distribution of AlbinoLevels among all edited lines was summarized in Figure 2E. All selected CRISPR-edited lines were classified according to albino phenotype severity (AlbinoLevel 0, 1, or 2), and detailed information for each line was provided in Supplementary Table S3, including genotyping profiles, the presence or absence of wild-type alleles, and the occurrence of albino branches. A trend was observed where frameshift mutations and larger deletions were more frequently associated with albino phenotypes (AlbinoLevel 1), although this association was not absolute. Conversely, in-frame mutations and base substitutions mostly resulted in green phenotypes, suggesting a non-deterministic relationship between mutation type and phenotypic expression.

### 2.3. RNA-Seq Data Quality Assessment and Global Transcriptomic Overview

**Figure 3:**
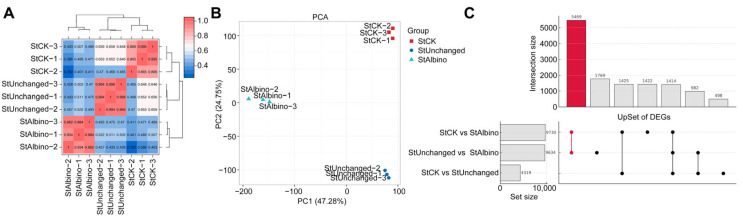
Global transcriptional variation and differential gene expression patterns among albino, non-albino, and wild-type potato samples. (**A**) Sample-to-sample correlation heatmap based on Pearson’s correlation coefficients (r). (**B**) Principal component analysis (PCA) showing clear separation of StCK, StUnchanged, and StAlbino samples, with tight clustering of biological replicates. (**C**) UpSet plot illustrating DEG intersections among the three comparisons. The highlighted intersection indicates the Albino Core Set.

To assess the transcriptomic consequences of *pds* gene editing, we performed RNA-seq analysis on albino seedlings (StAlbino) and phenotypically unaltered green tissues (StUnchanged) from the same DM1-18 edited line, with wild-type tissues (StCK) as controls. Each group included three biological replicates. High-throughput sequencing generated high-quality datasets, with detailed metrics summarized in Supplementary Table S4. Clean reads aligned to the reference genome with mapping efficiencies ranging from 85.09% to 88.70% (Supplementary Table S5), confirming the suitability of the data for downstream analyses. Pearson’s correlation coefficients (*r*) revealed high within-group reproducibility and robust separation among the three sample groups (Figure 3A,B).

Transcriptome-wide read alignment also enabled refinement of gene structural annotations in the DM v8.1 reference genome. Based on continuous read support, untranslated regions (UTRs) were updated for 5876 genes (Supplementary Table S6), and 2262 novel genes were identified from transcript assemblies, of which 1193 received functional annotations (Supplementary Tables S7 and S8). Notably, annotation inconsistency was observed at the *pds* locus. Comparative analysis with *S. stenotomum* v2.1 and NCBI reference sequences suggested that the existing DM v8.1 model represented a composite or chimeric annotation, with only the downstream 15 CDSs corresponding to the true exonic region (Supplementary Figure S3). Read coverage analysis supported this correction: both upstream and downstream regions showed similar expression in StCK and StUnchanged samples, but the authentic *pds* exons exhibited marked reduction in StAlbino, consistent with functional disruption of the target gene.

To investigate transcriptomic shifts associated with the albino phenotype, we performed pairwise comparisons across wild-type (StCK), non-albino transgenic (StUnchanged), and albino (StAlbino) samples. Albino tissues exhibited widespread transcriptional reprogramming, with over 9000 differentially expressed genes (DEGs) in comparisons against both wild-type and non-albino transgenic controls (Supplementary Figure S4). In contrast, only 4319 DEGs were detected between wild-type and non-albino transgenics, suggesting that *pds* knockout in the absence of visible albinism was associated with more limited transcriptomic changes, potentially reflecting partial buffering or compensatory regulation. To delineate phenotype-associated signatures, we defined two DEG subsets based on intersection analysis (Figure 3C, Supplementary Table S9): an Albino Core Set reflecting genes specifically dysregulated in albino tissues, and a Non-Albino Specific Set representing transgenic effects uncoupled from visible whitening.

### 2.4. Functional Enrichment Revealed Distinct Transcriptomic Reprogramming Between Partial and Complete Albinism

**Figure 4:**
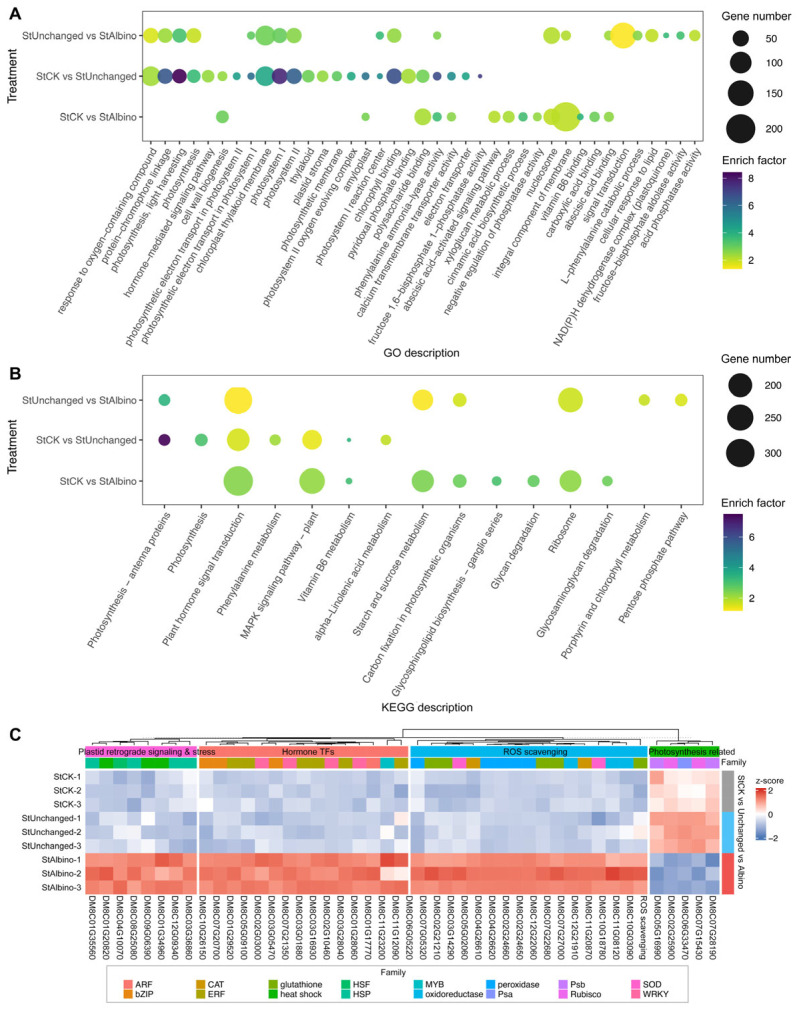
Functional enrichment and expression profiling of DEGs among StCK, StUnchanged, and StAlbino. GO (**A**) and KEGG (**B**) analysis of the DEGs in the comparison groups of StCK vs. StAlbino, StCK vs. StUnchanged and StUnchanged vs. StAlbino. (**C**) Heatmap of representative genes across three sample groups. Values were row-standardized z-scores (blue = downregulated, red = upregulated), illustrating consistent downregulation of photosynthetic genes in StAlbino and relative upregulation of genes involved in stress response, hormone signaling, and structural processes.

To characterize process-level transcriptional changes, we applied Gene Ontology (GO) over-representation analysis (ORA) and gene-set enrichment analysis (GSEA) to full-rank gene lists for StCK vs. StUnchanged and StCK vs. StAlbino across BP/CC/MF domains and KEGG pathways, controlling the false discovery rate at FDR < 0.05 (Figure 4A,B, full results in Supplementary Tables S10–S13). Guided by these enrichments, we highlighted four recurrent, biologically coherent categories, which were photosynthesis/primary carbon metabolism, ROS scavenging, plastid stress/retrograde signaling, and hormone-responsive transcription factors, and visualized expression with row-standardized heatmaps and gene-wise distribution plots (Figure 4C, Supplementary Figure S5).

In StCK vs. StUnchanged, enrichment patterns indicated functional transitions associated with partial leaf albinism. Photosynthesis-related processes, including photosynthesis, light harvesting, photosystem I, and protein-chromophore linkage, were strongly enriched, with corresponding CC terms dominated by chloroplast thylakoid membrane and MF terms by chlorophyll binding. These results indicated substantial attenuation of photosynthetic modules during partial albinism. Consistently, photosynthesis and light-harvesting gene sets were negatively enriched (NES < 0), with coordinated downregulation of key components such as rbcS, PsaE, PSII 10 kDa polypeptide, and oxygen-evolving enhancer proteins, reflecting reduced light capture and photochemical efficiency. In parallel, detoxification- and hormone signaling-related gene sets were positively enriched (NES > 0), accompanied by upregulation of class III peroxidases, glutathione S-transferases, Fe/Mn-superoxide dismutases, and induction of ERF/AP2, WRKY, and bZIP transcription factors, consistent with early activation of stress-responsive regulatory programs triggered by pigment deficiency.

By contrast, StCK vs. StAlbino exhibited broader and more extensive transcriptional shifts consistent with the albino phenotype. Differentially expressed genes shifted from photosynthesis-related functions toward cell wall biogenesis/remodeling, hormone signaling, secondary metabolism, polysaccharide turnover, and plastid stress/chaperone pathways. BP terms highlighted cell wall biogenesis and abscisic acid-activated signaling, while MF terms were dominated by vitamin B6 binding, phenylalanine ammonia-lyase activity, and glycogen phosphorylase activity. GSEA confirmed this transition, showing positive enrichment of ribosome and plant-type cell wall organization with persistent depletion of photosynthetic programs. Consistently, KEGG pathways related to plant hormone signaling, starch and sucrose metabolism, carbon fixation, vitamin B6 metabolism, and glycosphingolipid biosynthesis were significantly enriched. Together, these patterns suggested that complete albinism was associated with pronounced disruption of chloroplast-associated functions, elevated oxidative and plastid stress responses, altered plastid-nucleus communication, and reorganization of carbon allocation away from photosynthetic output toward structural and defense-related processes.

### 2.5. WGCNA Module Identification and Phenotypic Association

**Figure 5:**
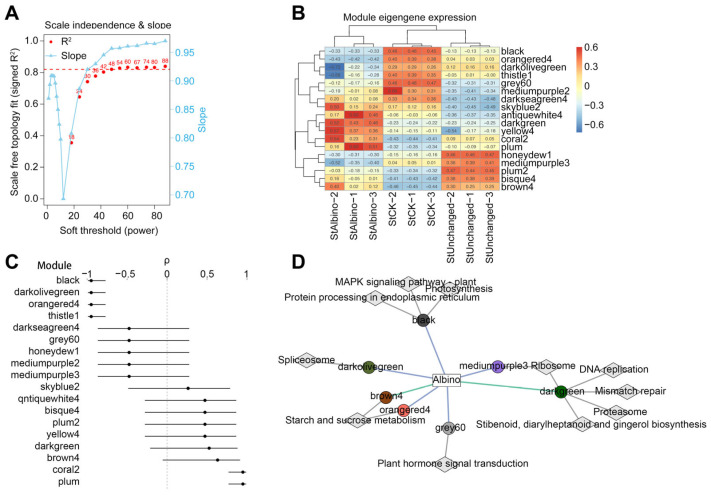
Identification of WGCNA co-expression modules and their association with albino phenotype severity. (**A**) Scale-free topology fitting and soft-threshold power selection. (**B**) Correlation between module eigengenes and the albino phenotype gradient. (**C**) Monotonic expression trends of selected module eigengenes across CK, Unchanged, and Albino samples. (**D**) Module-pathway-phenotype network showing functional associations between WGCNA modules and KEGG-enriched pathways in relation to increasing albino severity.

To investigate co-expression patterns associated with *pds*-knockout-induced albinism, we built a weighted gene co-expression network from variance-stabilized counts (DESeq2-vst) of nine samples across StCK, StUnchanged, and StAlbino. A signed adjacency (biweight mid-correlation) was transformed to a (signed) topological overlap matrix for hierarchical clustering. Using a soft-threshold power β = 74, the network met the scale-free criterion (fit index R^2^ = 0.83; mean connectivity K = 242; slope = −0.992; Figure 5A; Supplementary Table S14). Modules were identified by dynamic tree cut (minimum module size = 30) and merged by module eigengene (ME) dissimilarity (1 − cor) = 0.05 (i.e., ME correlation ≥ 0.95), yielding 18 modules.

To relate modules to phenotype, we defined an ordinal bleaching score (StCK = 0, StUnchanged = 1, StAlbino = 2) and correlated MEs with the score (Pearson, Benjamini-Hochberg adjusted; Spearman reported in Supplementary Table S15). Nine modules were significant after correction (Figure 5B). The strongest negative association was MEorangered4 (r = −0.998, *p* = 2.25 × 10^−9^), followed by MEblack (r = −0.996, *p* < 0.001) and MEthistle1 (r = −0.921, *p* = 0.0004), indicating progressive down-shift with increasing bleaching severity. In contrast, MEcoral2 (r = 0.962, *p* = 3.46 × 10^−5^), MEplum (r = 0.925, *p* = 0.0003), MEdarkgreen (r = 0.855, *p* = 0.014), and MEbrown4 (r = 0.774, *p* = 0.038) were positively associated, consistent with phenotype-associated up-regulation. Ordered-group trend testing (Jonckheere-Terpstra, BH-FDR < 0.05) confirmed that most of the nine significant modules exhibited monotonic expression changes across the CK, Unchanged, and Albino groups (Figure 5C). MEorangered4/MEblack/MEthistle1/MEdarkolivegreen progressively decreased, whereas MEcoral2/MEplum/MEdarkgreen/MEbrown4 progressively increased. MEgrey60 was negatively correlated (r = −0.750, *p* = 0.041) but showed a V-shaped mean (Unchanged minimum, slight rebound in Albino). Overall, module-level monotonicity supported their association with the progression of leaf albinism.

### 2.6. Functional Enrichment of Trait-Associated Modules Revealed Coordinated Metabolic and Signaling Shifts

We next assessed the biological relevance of phenotype-associated modules by KEGG over-representation analysis (BH-FDR; background = all expressed genes; Supplementary Table S16), integrating pathway enrichments with module eigengene (ME) expression trends (Figure 5D). To anchor each module to the albino phenotype, representative high-kME genes were further examined across StCK, StUnchanged, and StAlbino (Supplementary Figure S6). Genes in MEdarkgreen were predominantly upregulated with increasing albinism and were enriched in DNA replication, ribosome, proteasome, mismatch repair, and stilbenoid/diarylheptanoid/gingerol biosynthesis (*q* = 0.014), with marginal enrichment in cutin, suberin, and wax biosynthesis (*q* = 0.0548). Representative genes involved in secondary metabolism and cuticular/lipid barrier formation (e.g., acyltransferases and fatty-acid elongation/export factors) showed coordinated induction with bleaching severity, suggesting concomitant activation of metabolic renewal processes and physical barrier-related pathways during albinism progression.

Among negatively associated modules, MEorangered4 was significantly enriched for starch and sucrose metabolism (*q* = 3.38 × 10^−4^), indicating repression of carbohydrate metabolism during albinism. Most module genes showed monotonic downregulation along the bleaching gradient, including carbohydrate metabolic enzymes (e.g., β-amylase, glycosyltransferases, glycosyl hydrolases) and multiple resistance-related genes. In contrast, a small subset was upregulated, primarily genes involved in sugar signaling and alternative carbon metabolism (e.g., trehalose-6-phosphate phosphatase, pfkB-family kinases). This pattern indicated that, despite broad suppression of plastid-dependent carbon assimilation and basal defense transcription, selected sugar-metabolic and carbon reallocation components remained transcriptionally responsive within MEorangered4. Notably, although MEbrown4 was overall positively associated with bleaching, several of its representative genes showed downregulation and were likewise enriched in starch and sucrose metabolism, particularly phosphorylase-related functions, indicating that distinct functional branches of carbohydrate metabolism were partitioned across different co-expression modules while remaining consistent with the overall repression of carbon metabolism during albinism.

Also, MEblack showed sustained downregulation with increasing albinism and was enriched for MAPK signaling and protein homeostasis pathways (*q* = 0.0379), consistent with attenuated stress-signaling capacity in albino tissues. The module comprised numerous endoplasmic reticulum protein folding and quality-control components (e.g., HSP70/HSP20, DnaJ, calreticulin), ubiquitin-conjugating enzymes, and UBX-domain proteins, together with receptor-like kinases, serine/threonine kinases, MAPK cascade components, and stress- or hormone-responsive transcription factors (WRKY, ERF/AP2, bZIP), all coordinately downregulated. In parallel, MEgrey60 was significantly enriched for plant hormone signal transduction (*q* = 0.0198) and exhibited pervasive downregulation across the bleaching gradient. This pathway encompassed core regulators of auxin, brassinosteroid, gibberellin, and ethylene signaling (e.g., ARFs, auxin-induced proteins, BES1/BZR1 homologs, GRAS family members, PP2C phosphatases, and multiple protein/receptor-like kinases), as well as MYB, bHLH, and MYC transcription factors, indicating broad attenuation of hormone perception, signaling, and downstream transcriptional regulation in albino tissues. Collectively, albinism was characterized by layered transcriptional repression patterns, encompassing carbon metabolism (MEorangered4), stress signaling and protein homeostasis (MEblack), and hormone-integrative developmental regulation (MEgrey60).

Cross-validation with DEG sets (Fisher’s exact test, BH-FDR) further quantified module-phenotype links. MEdarkgreen significantly overlapped the Albino core (AB-only) set (OR = 23.65, 95% CI 21.54–25.99, FDR < 10^−10^). MEorangered4 was enriched in the ABC (global-responsive) set (OR = 5.54, 95% CI 4.72–6.50, FDR = 5.36 × 10^−85^). MEbrown4 (OR = 23.18, 95% CI 14.79–37.27, FDR = 1.67 × 10^−50^), MEgrey60 (OR = 29.32, 95% CI 24.43–35.32, FDR = 9.94 × 10^−60^), and MEblack (OR = 17.44, 95% CI 15.27–19.92, FDR < 10^−10^) overlapped the AC-only (transgenic-background) set. Gene-level expression patterns were concordant with pathway-level enrichments within each module. In photosynthesis-depleted modules (e.g., MEorangered4 and antenna/thylakoid-enriched sets), representative LHC, PSI/PSII subunits, and RBCS showed consistent downregulation in albino tissues, matching the negative enrichment of photosynthesis and light-harvesting pathways. Modules enriched for detoxification and plastid stress exhibited induction of ROS-scavenging enzymes (CAT/SOD/APX) and chaperone families (HSPs), consistent with their pathway annotations. Hormone signal transduction-enriched modules contained ABA/JA-responsive regulators and downstream transcription factors (ERF/WRKY/bZIP) whose expression changes were concordant with pathway-level trends. In addition, MEbrown4 showed enrichment for starch and sucrose metabolism accompanied by coordinated regulation of sucrose-utilization genes, consistent with its module-level enrichment profile.

Together, with KEGG enrichment results, these observations indicated a hierarchically organized transcriptional response underlying *pds* knockout-induced albinism. MEdarkgreen module was associated with albino-linked activation of secondary metabolism and physical barrier related processes and showed strong overlap with the Albino Core (AB-only) gene set. MEorangered4 corresponded to a carbohydrate-metabolic execution layer that was broadly downregulated during albinism, consistent with reduced photosynthate availability and its enrichment in the global-responsive (ABC) gene set. In contrast, MEblack, MEgrey60, and MEbrown4 were predominantly associated with the transgenic-background (AC-only) gene set and exhibited coordinated downregulation of stress signaling, protein homeostasis, and hormone-integrative regulatory components.

## 3. Discussion

### 3.1. Albino Phenotype Is Associated with Broad Transcriptional and Metabolic Alterations

**Figure 6:**
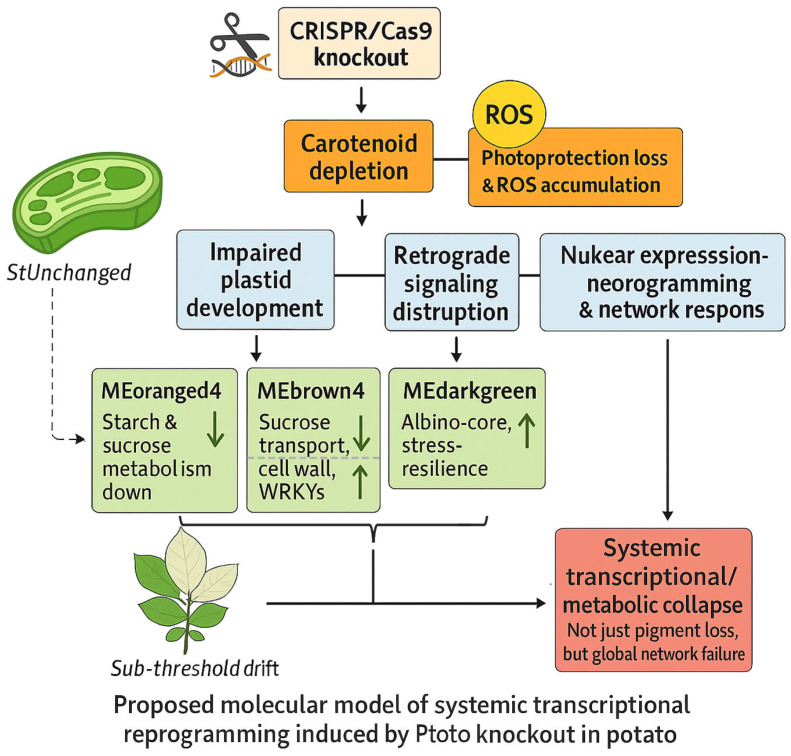
Schematic model of the molecular cascade triggered by *pds* knockout. Loss of carotenoids leads to plastid dysfunction, ROS accumulation, and impaired retrograde signaling. These changes trigger nuclear gene reprogramming, with suppressed photosynthesis and activated stress-response pathways, ultimately resulting in the albino phenotype. The darkgreen arrows indicate upregulation or downregulation of functional genes in the module.

The albino phenotype induced by *pds* knockout in potato is not simply a pigment deficiency syndrome, but it is accompanied by widespread transcriptional and metabolic changes [9,17]. While PDS, a key enzyme in the carotenoid biosynthesis pathway, has traditionally served as a visual marker for editing efficiency [18], our transcriptomic and co-expression analyses revealed coordinated alterations across multiple regulatory and metabolic pathways (Figure 6). Specifically, albino lines exhibited significant downregulation of genes involved in primary carbon metabolism (e.g., module MEorangered4), alongside activation of pathways related to secondary metabolism and cell wall remodeling (e.g., MEdarkgreen). These expression patterns resembled features of canonical retrograde signaling responses observed in plastid-defective or photobleached mutants, where plastid dysfunction initiated nuclear transcriptional reprogramming to cope with oxidative stress and metabolic imbalance [19,20,21]. Mechanistically, loss of *pds* may disrupt plastid-to-nucleus communication by simultaneously depleting carotenoid-derived apocarotenoids and elevating ROS levels, both of which serve as critical plastid-derived signals [14,22]. This disruption may compromise the coordination of nuclear gene expression with plastid function, thereby contributing to gene network reorganization and stress response activation [23].

We proposed a model in which *pds* knockout leads to apocarotenoid depletion and ROS accumulation, thereby engaging plastid-to-nucleus (retrograde) signaling and driving coordinated changes in nuclear gene expression associated with the albino phenotype. This model extended the interpretation of albinism beyond pigment loss alone, suggesting that it reflects impaired regulatory buffering and altered metabolic balance under disrupted photosynthetic conditions. The phenotypic shift from StUnchanged to StAlbino may represent a regulatory transition point at which plastid dysfunction becomes irreversible [23,24]. While our transcriptome-wide evidence supported this framework, further validation through ROS quantification, apocarotenoid profiling, and monitoring of canonical retrograde markers (GUN1, ABI4, GLK1) will be necessary to confirm the causality and to refine our understanding of plastid-nucleus communication in genome-edited systems.

### 3.2. Passive Damage or Programmed Response in Albino Plants

Module behavior is more consistent with an organized regulatory response than with simple passive damage. As shown in Figure 6, the regulatory architecture underlying the albino phenotype involved multi-layered transcriptional modules that were jointly associated with photosynthetic dysfunction through diverse metabolic and signaling routes. Gene-level increased in CAT/SOD/APX and HSP families within stress-enriched modules (MEgrey60/MEblack/MEdarkgreen) aligned with elevated oxidative load and proteostasis demands in StAlbino, whereas photosynthetic subunits (e.g., RBCS, PsaE, PSII 10-kDa polypeptide/oxygen-evolving enhancer) decreased concordantly in MEorangered4. Notably, modules with hormone-signal signatures (e.g., plant hormone signal transduction in MEgrey60) show induced ABA/JA-responsive TFs (ERF/WRKY/bZIP), which was compatible with hormone-mediated rewiring during plastid stress. These transcriptional circuits resemble previously identified stress-induced hubs involved in orchestrating metabolic and hormonal responses under abiotic constraints [25,26,27]. Cross-validation with phenotype-defined DEG sets (Section 2.6) further linked MEdarkgreen to the Albino Core Set, suggesting its potential utility as a cross-genotype biomarker for chloroplast dysfunction. Its consistent co-expression patterns aligned with recent advances in predictive network biology, where conserved transcriptional modules were used to infer plant response types across diverse genetic and environmental backgrounds [28,29].

We cautioned that background-associated modules may capture transgenic/vector or culture effects. Modules such as MEgrey60 and MEblack, although not directly linked to pigment metabolism, showed expression patterns aligned with the transgenic background and were enriched in hormone signaling, protein processing, and environmental response pathways. This suggested that these modules may be associated with background-associated regulatory variation, involving hormone signaling, protein processing, and environmental response pathways [30]. Nonetheless, the convergence on redox/hormone/cell-wall programs was consistent with conserved stress circuitry seen in other crops [31,32,33]. Importantly, due to the absence of empty-vector controls and Cas9 expression profiling in the present study, the background-associated modules identified here cannot be conclusively attributed to vector-driven regulation and should be interpreted cautiously as transgenic background-related transcriptional effects.

Importantly, StUnchanged samples showed module drift (e.g., MEdarkgreen, MEgrey60) despite lacking visible bleaching, indicating that the absence of phenotype does not equate to the absence of molecular perturbation [34]. Instead, StUnchanged samples may represent an intermediate regulatory state, positioned near the threshold of transcriptional instability [35,36]. This raised the possibility of subtle editing escape, vector-related expression artifacts, or microenvironmental influences triggering weak but detectable molecular responses [37,38]. Such observations challenged the binary interpretation of gene-editing outcomes and highlighted the importance of transcriptomic profiling even in phenotypically neutral cases [39]. Far from being “inactive”, these samples could unveil early warning signatures of unintended or sub-threshold biological effects [40,41]. Integrating these subtle transcriptional changes into network-based analytical frameworks could improve the evaluation of off-target risks, transcriptional compensation, and hidden regulatory feedback loops following genome editing [42,43], and supported targeted validation, such as ROS quantification, apocarotenoid profiling, and monitoring of canonical retrograde markers (e.g., GUN1, ABI4, GLK1) to refine causal relationships.

### 3.3. Rethinking pds-Based Visual Screening Tools

While the knockout of the *pds* gene offers a highly visible albino phenotype for screening genome-edited plants, our results, as well as emerging transcriptomic evidence, highlighted heterogeneity that challenges phenotype-only screening. Several edited lines without noticeable bleaching nevertheless exhibited clear transcriptional changes, including module drift and network rewiring. This heterogeneity raised concerns about both false negative and false positive outcomes when using *pds*-induced albinism as a primary selection tool.

First, “normal-looking” edited plants (StUnchanged) can harbor significant transcriptional shifts, including module drift and TF/chaperone induction, risking false negatives if bleaching alone was used. For instance, expression shifts in modules such as MEgrey60 and MEdarkgreen suggested that lack of visible phenotype does not equate to absence of editing- or background-related impact. Thus, the screening system may suffer from under-detection of true edits (false negatives) when only visible bleaching was used as the criterion. Recent studies in other crops had similarly shown that plants lacking visible morphological changes can still harbor significant transcriptomic responses to editing or tissue-culture stress [44,45].

Second, false positives may also be a risk: some lines showing visible bleaching might result not just from target gene knockout but from vector integration effects, somaclonal variation, or tissue-culture-induced stress. Hence, the specificity of the *pds* bleaching marker may be compromised by background noise or off-target responses. Reviews of genome-editing pipelines caution that phenotypic markers, while convenient, must be interpreted in light of molecular data to avoid misclassification of editing outcomes [36,46]. We, therefore, recommend integrating visible phenotype with molecular signatures (e.g., module-level readouts (MEbrown4/MEdarkgreen); early marker genes (CAT/SOD/APX; HSP20/60/90; ERF/WRKY/bZIP); photosystem subunits as internal negatives) to improve sensitivity/specificity. Such multi-dimensional validation aligns with emerging best practices in plant genome editing [40,41,47].

Finally, our potato dataset represents, to our knowledge, one of the first comprehensive transcriptome-wide analyses of *pds*-induced albinism in this crop, filling a gap with cross-species relevance and offering module- and gene-level markers for future screening pipeline design. For future work, developing additional or alternative visual-reporter systems that respond robustly and early to targeted edits may enhance the reliability of high-throughput screening, including fluorescent reporters, metabolite-based colorimetric assays, or rapid molecular sensors integrated into editing vectors. While *pds*-induced bleaching remains valuable for rapid preliminary screening, its limitations underscore the need for refined and integrated validation frameworks to fully capture the downstream impact of editing events and potential background perturbations [37,42].

## 4. Materials and Methods

### 4.1. Tissue Culture Conditions and CRISPR/Cas9 Knockout Vector Construction

Diploid cultivated potato (*Solanum phureja*, DM1) was maintained on MS medium supplemented with 30 g/L sucrose and 0.7% (*w*/*v*) agar (pH 5.8) at 22–24 °C under a 16 h light/8 h dark photoperiod (~2000 lx). Explants bearing axillary buds (stem segments) were subcultured every 6–8 weeks.

Based on the Sanger-verified *pds* sequence, sgRNA candidates were designed in CRISPOR (organism/assembly: SolTub_3.0; nuclease: SpCas9, PAM = NGG). Candidates were ranked by Rule Set 2 on-target activity and MIT/CFD specificity scores [48]. We retained guides meeting standard quality filters: GC 40–70%, no ≥ 4-nt homopolymers, unique 10–12-nt PAM-proximal seed, and no predicted exonic off-targets with ≤ 2 mismatches across the genome. Repetitive regions and known variants in SolTub_3.0 were masked, and guides spanning splice junctions or polymorphic sites were excluded. To maximize loss-of-function, targets were restricted to early coding exons and positions likely to yield frameshift indels. The guide 8964-JC (protospacer 5′-TCACAAACCGATACTACTGG-3′, exon 4 of *pds*) satisfied these criteria, showing high predicted on-target activity and high specificity in CRISPOR, with no exonic off-targets at ≤ 2 mismatches. The target fragment was amplified with primers cas9-8964-F/cas9-8964-R (Supplementary Table S1) using PCR (95 °C 30 s; 50 °C 15 s; 68 °C 15 s for 35 cycles; final extension 72 °C 10 min). After gel purification with TaKaRa MiniBEST Gel Extraction Kit (TaKaRa Bio, Dalian, China), the amplicon was inserted into the psgR-Cas9-AT vector by homologous recombination cloning (New England Biolabs, Ipswich, MA, USA).

To assemble the final binary construct, the recombinant segment (guide cassette + vector backbone) was re-amplified with 2300cas-F/2300cas-R (PCR: 95 °C 5 min; then 94 °C 30 s, 55 °C 30 s, 72 °C 30 s for 30 cycles; 72 °C 10 min). The pCAMBIA2300-cas9 vector was digested with HindIII and SmaI (Takara Bio, Kusatsu, Japan), gel-purified, and ligated with the recombinant insert (T4 DNA ligase, New England Biolabs, Ipswich, MA, USA). Ligation mixtures were transformed into chemically competent *E. coli* DH5α; colonies selected on kanamycin plates were screened by colony PCR with 2300CAS-F/8964-JC-R and confirmed by Sanger sequencing (Sangon Biotech Co., Ltd., Shanghai, China). Plasmid DNA from sequence-verified clones was prepared using Plasmid Mini Kit I (OMEGA, Cat. D6943-01), yielding the working construct pCAMBIA2300-CAS9-8964 for subsequent experiments.

### 4.2. Agrobacterium-Mediated Transformation of Potato

The binary construct pCAMBIA2300-CAS9-8964 was introduced into Agrobacterium tumefaciens GV3101 by freeze–thaw method; transformants were selected on LB agar containing kanamycin 50 mg/L and rifampicin 50 mg/L at 28 °C for 2–3 days and verified by colony PCR. Sterile in vitro stem segments (~2 cm) were immersed in the resuspended Agrobacterium suspension for 10 min, blotted dry on sterile filter paper, and transferred to co-cultivation medium (MS + IAA 1.0 mg/L + GA_3_ 0.2 mg/L + ZT 2.0 mg/L + 6-BA 0.5 mg/L + 100 uM AS + sucrose 30 g/L + agar 7.4 g/L, pH 5.8) in the dark for 2–3 days. Explants were then transferred to shoot-induction/selection medium (same hormones, plus kanamycin 100 mg/L and Timentin 300 mg/L; pH 5.8) with medium renewal every 2 weeks to induce resistant buds. Adventitious shoots were excised to rooting medium (MS + kanamycin 100 mg/L + Timentin 300 mg/L + sucrose 30 g/L + agar 7.4 g/L, pH 5.8) until well-developed roots formed.

### 4.3. Identification of Positive Transformants and Phenotypic Assessment

Genomic DNA was isolated from regenerated plantlets by the SDS protocol, and the *pds* target region was first assayed by PCR using primers 8964-F/8964-R (Supplementary Table S1). PCR-positive transformants were then subjected to on-target genotyping: the *pds* locus was amplified with PDStec1-F/PDStec1-R, amplicons were Sanger-sequenced, and sequences were aligned to the wild-type reference (StCK) to call edits. Plants carrying any detectable indel at the *pds* target were called edited positives; per-line targeting efficiency = (edited plants/tested plants) × 100% [49]. Edited lines and wild-type controls were sub-cultured twice under identical conditions, incidence and severity scoring (AlbinoLevel 0/1/2) by two blinded raters, with disagreements reconciled by consensus [13].

### 4.4. TIDE Analysis of Editing Efficiency

To assess the CRISPR/Cas9-induced mutation profiles at the target site, Sanger sequencing chromatograms from PCR-amplified genomic DNA were subjected to Tracking of Indels by DEcomposition (TIDE) analysis (https://tide.nki.nl/) [50]. Sequences were aligned using a ±30–50 bp window surrounding the predicted Cas9 cleavage site. TIDE deconvoluted the mixed sequencing traces to estimate the spectrum and relative frequencies of insertion and deletion events. The output included the percentage of wild-type alleles, the proportion of frameshift mutations, and the distribution of specific indel sizes. Editing events were further manually validated based on sequence alignment and chromatogram inspection.

### 4.5. Transcriptome Sequencing and Differential Expression

To assess transcript-level effects associated with the co-occurrence of albino and non-albino tissues within the same edited line, we sampled the CRISPR/Cas9-edited line DM1-18 (non-albino leaves, StUnchanged; albino seedling tissue, StAlbino) alongside wild-type non-transgenic plants (StCK) as controls (*n* = 3 biological replicates per group). Total RNA was extracted with TRIzol (Invitrogen, Carlsbad, CA, USA) followed by DNase I (Takara Bio, Kusatsu, Japan) treatment; integrity was verified using a Bioanalyzer 2100 (Agilent Technologies, Santa Clara, CA, USA) (RIN ≥ 7.0) [51]. Poly(A)+ libraries were constructed and sequenced on an Illumina PE150 platform (≥ 6–8 Gb per sample). Reads were trimmed with Trimmomatic v0.39 [52] and aligned to the Solanum tuberosum DM8.1_v2 reference using HISAT2 v2.2.1 [53]. Reference-guided assembly and quantification were performed with StringTie v2.2.1 against the GTF to obtain FPKM values [54]. Raw gene-level counts from prepDE.py were analyzed with DESeq2 v1.46.0 (R v4.4.x) [55]. Low-count genes (sum < 10 across libraries) were filtered; size factors were estimated by the median-ratio method and dispersions fit with the default parametric trend. Pairwise contrasts (StCK vs. StAlbino, StUnchanged vs. StAlbino, StCK vs. StUnchanged) used Wald tests with Benjamini–Hochberg correction; DEGs were defined as |log_2_FC| ≥ 1 and FDR < 0.01. Variance-stabilized counts supported PCA and replicate concordance heatmaps (pheatmap v1.0.12) [56]. Novel gene predictions were annotated by DIAMOND searches against NR/Swiss-Prot/COG/KOG/KEGG, GO via InterProScan, and domains via HMMER against Pfam [57,58,59].

Intersection patterns were summarized by an UpSet framework and used to define two high-confidence sets. The Albino Core Set comprises genes that were significantly and concordantly regulated in both comparisons involving StAlbino, while remaining non-significant between StCK and StUnchanged (FDR ≥ 0.10 and |log_2_FC| < 0.3). The Non-Albino Core Set includes genes significant only in StCK vs. StUnchanged and non-significant in either comparison that includes StAlbino. To enhance robustness, we further required (i) at least one group mean FPKM ≥ 1, (ii) within-group coefficient of variation (three replicates) ≤ 0.5, and (iii) exclusion of outliers identified by median ± 3 × MAD. For confirmatory groupwise testing at the single-gene level, we applied a Kruskal–Wallis test followed, where appropriate, by pairwise Wilcoxon rank-sum tests; when normality (Shapiro-Wilk) was satisfied, one-way ANOVA and Welch’s t-tests were used instead. All multiple testing was corrected by the Benjamini–Hochberg method, and final “core” calls required an overall FDR < 0.05, significant target pairwise contrasts (FDR < 0.05), and maintenance of the “no-change” channel (FDR ≥ 0.10 with |log_2_FC| < 0.3).

Enrichment used GO/KEGG over-representation (clusterProfiler; background = expressed genes) and GSEA on full-rank lists (signed Wald statistic), reporting NES and FDR (q < 0.05) for significance; gene-length bias checks were cross-validated with goseq (Wallenius). All analyses and plotting were done in R (tidyverse, DESeq2, clusterProfiler, goseq/KOBAS, pheatmap, ComplexHeatmap, ggplot2, ggpubr, ggbeeswarm), with FPKM (log_2_(FPKM + 1)) for visualization and counts for statistical modeling.

Guided by enrichment results and the physiology of albinism, we focused downstream pattern analysis on four coherent functional classes: photosynthesis/primary carbon metabolism, ROS scavenging, plastid retrograde/stress-responsive factors, and hormone-related transcription factors. For visualization, heatmaps were drawn, and gene-wise distributions across groups were depicted by a violin. A Kruskal–Wallis test was used for the overall group difference. If the omnibus test was significant (BH-adjusted *q* < 0.05), we ran the three planned pairwise comparisons with Welch’s two-sided t-test when group data were approximately normal (Shapiro-Wilk *p* > 0.05) and variances comparable; otherwise, we used the Wilcoxon rank-sum test. All pairwise *p*-values were Benjamini–Hochberg adjusted, and only significant results (*q* < 0.05) were annotated in plots. All analyses were conducted in R using tidyverse, DESeq2, ComplexHeatmap, ggplot2, ggpubr, rstatix, and ggbeeswarm.

### 4.6. Co-Expression Network Analysis

For co-expression analysis, we used the VST matrix from DESeq2::vst. WGCNA v1.72-1 was run on the top 50% most variable genes (by VST variance), excluding genes with mean VST < 1 [60]. Samples were hierarchically clustered to assess outliers (none removed). An unsigned network was built; the soft-thresholding power (β) was chosen via pickSoftThreshold to maximize scale-free topology fit while retaining adequate mean connectivity. The adjacency matrix was computed, transformed to a TOM, and modules were detected by dynamic tree cut (minModuleSize = 30), then merged at mergeCutHeight = 0.05. Module eigengenes (MEs) were correlated (Pearson’s *r*) with a pseudo-trait representing bleaching severity (StCK = 0, StUnchanged = 1, StAlbino = 2); significance was assessed with BH-adjusted *p*-values (FDR < 0.05). Monotonic trends across the ordered groups were additionally evaluated using Jonckheere–Terpstra tests. Hub genes were defined per module as the top 10 by intramodular connectivity (kWithin); for reporting, we also provide ME-based connectivity (kME) as a complementary ranking when relevant.

Functional enrichment was performed with clusterProfiler v4.8.2 [61], using both GO/KEGG over-representation analysis (ORA; reporting enrichment factor and FDR) and GSEA on full-rank gene lists (reporting normalized enrichment score, NES, and FDR). Unless otherwise stated, the gene universe for ORA was the set of expressed genes (post-filtering) to control for detection bias; for module-focused ORA, each module was tested against the same expressed-gene background. Significance thresholds were *q* ≤ 0.05 for ORA and *q* ≤ 0.25 for GSEA (BH). TOM-based subnetworks (edge weight threshold on TOM > 0.2) were exported to Cytoscape v3.10.3 for visualization [62]. VST-scale expression trends of hub/representative genes across StCK/StUnchanged/StAlbino were plotted as needed to align gene-level patterns with module- and pathway-level signals.

## 5. Conclusions

This study established a *pds*-targeted, visually scorable CRISPR/Cas9 platform in potato and provided a network-based analytical framework for interpreting *pds* knockout-induced albinism. With a transformation efficiency of 80.95%, we showed that bleaching was not a binary readout of “edited vs. non-edited” but was more consistent with the extent of functional disruption and the presence of within-plant chimerism. Transcriptome profiling revealed albino tissues underwent extensive transcriptional reprogramming (> 9000 DEGs versus both wild type and non-albino edited tissues), whereas edited tissues without visible bleaching differed more moderately from wild type (4319 DEGs), indicating distinct molecular divergence underlying phenotypic variation. WGCNA further organized this phenotype into coherent regulatory layers: module-trait associations were strong and mapped to distinct functional strata, including repression of a carbon-metabolic execution layer (MEorangered4, linked to starch/sucrose metabolism), attenuation of stress signaling/proteostasis regulation (MEblack, linked to MAPK/proteostasis), and down-shift of hormone-integrative developmental control (MEgrey60, linked to hormone signaling). In contrast, MEdarkgreen marked an albino-linked activation program with strong overlap with the Albino Core (AB-only) set, reflecting coordinated induction of cellular renewal and secondary metabolism/barrier-related processes. Collectively, our results repositioned *pds*-based bleaching from a simple visual marker to a quantifiable, modular systems response driven by plastid failure and loss of photosynthate supply, with downstream metabolic reallocation and signaling rewiring. This conceptual framework explained phenotypic heterogeneity in *pds*-edited materials and offered practical module/gene markers to complement phenotype-only screening in future potato editing pipelines.

## Data Availability

The transcriptome sequencing data supporting the findings of this study have been deposited in the NCBI Sequence Read Archive (SRA) under accession number PRJNA1358920. All other relevant data, including expression profiles and Supplementary Materials, are included within the article and its Supplementary Information Files. Additional data are available from the corresponding author upon reasonable request.

## Supplementary Materials

The following supporting information can be downloaded at: https://www.mdpi.com/article/10.3390/plants15010096/s1, Figure S1: PCR validation of sgRNA-construct assembly and transformant screening. (A) Amplification of the *pds* target region (sgRNA 8964 locus) from genomic DNA. (B) PCR amplification of the 8964 recombinant fragments used for vector assembly. (C) *E. coli* colony PCR screening of pCAMBIA2300-CAS9-8964 clones. (D) Agrobacterium tumefaciens GV3101 colony PCR verification of pCAMBIA2300-CAS9-8964; Figure S2: Representative Sanger sequencing chromatograms showing on-target editing patterns at the *pds* locus in potato; Figure S3: Visualization of CDS alignment and read mapping results of the *pds* gene; Figure S4: Volcano plots of differentially expressed genes (DEGs) from three pairwise comparisons (StCK vs. StAlbino, StUnchanged vs. StAlbino, and StCK vs. StUnchanged); Figure S5: Violin plots of representative genes across StCK, StUnchanged, and StAlbino; Figure S6: Expression patterns of representative high-kME genes across the bleaching gradient. Table S1: Primers used in this study; Table S2: High-confidence off-target candidates filtered by mismatch number, PAM type, CFD score, and genomic location; Table S3: Summary of genotyping and albino phenotype classification across CRISPR/Cas9-edited potato lines targeting *pds*; Table S4: Summary of sequencing data quality metrics from high-throughput transcriptome analysis; Table S5: Summary of sequencing read alignment statistics against the selected potato reference genome; Table S6: Results of gene structure optimization in the reference genome; Table S7: Annotation information of novel genes in GFF format; Table S8: Functional annotations of novel genes based on multiple databases; Table S9: Gene lists corresponding to intersections and unions in the UpSet analysis; Table S10: GO term enrichment analysis summary across pairwise comparisons; Table S11: KEGG pathway enrichment analysis summary across pairwise comparisons; Table S12: Gene Set Enrichment Analysis (GSEA) of GO terms across pairwise comparisons; Table S13: Gene Set Enrichment Analysis (GSEA) of KEGG terms across pairwise comparisons; Table S14: Soft-threshold Power Selection and Network Topology Parameters for WGCNA; Table S15: Correlation between module eigengenes and albino phenotype severity; Table S16: KEGG enrichment analysis results of genes within each module.
