# Supplementary figures and images for: CRISPR/Cas9-Mediated pds Knockout in Potato Reveals Network-Level Transcriptomic Reorganization Beyond Pigment Loss
Source: Plants (Basel). 2025 Dec 28;15(1):96. doi: 10.3390/plants15010096 (PMC12787435; doi:10.3390/plants15010096)

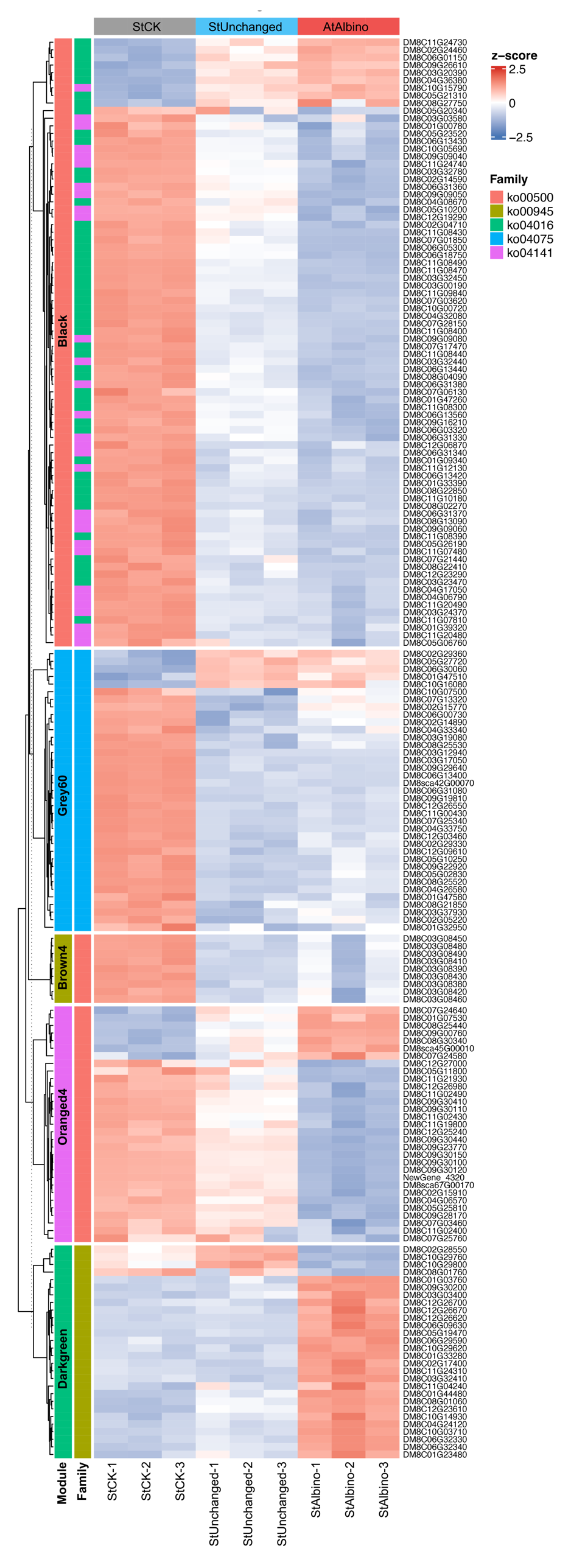

Supplement: Supplementary file 1 [file plants-15-00096-s001.zip › FigureS6m.tif]

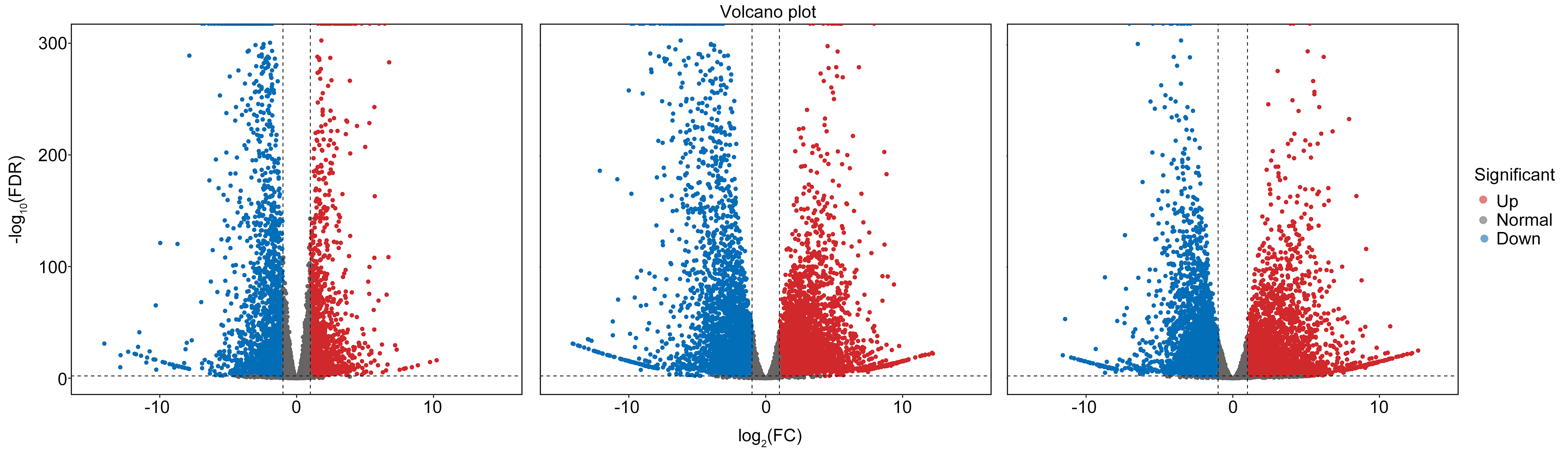

Supplement: Supplementary file 1 [file plants-15-00096-s001.zip › FigureS4m.tif]

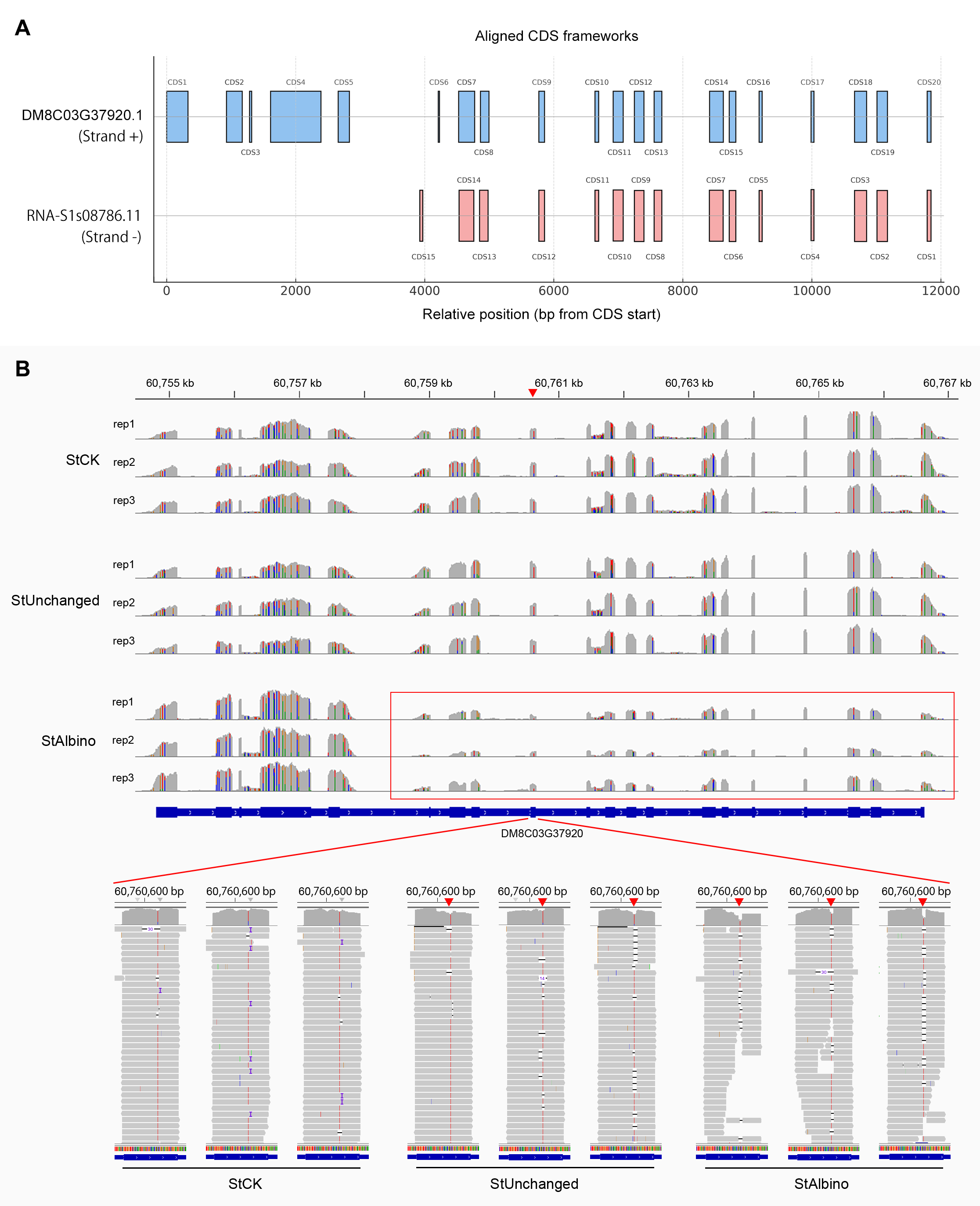

Supplement: Supplementary file 1 [file plants-15-00096-s001.zip › FigureS3m.tif]

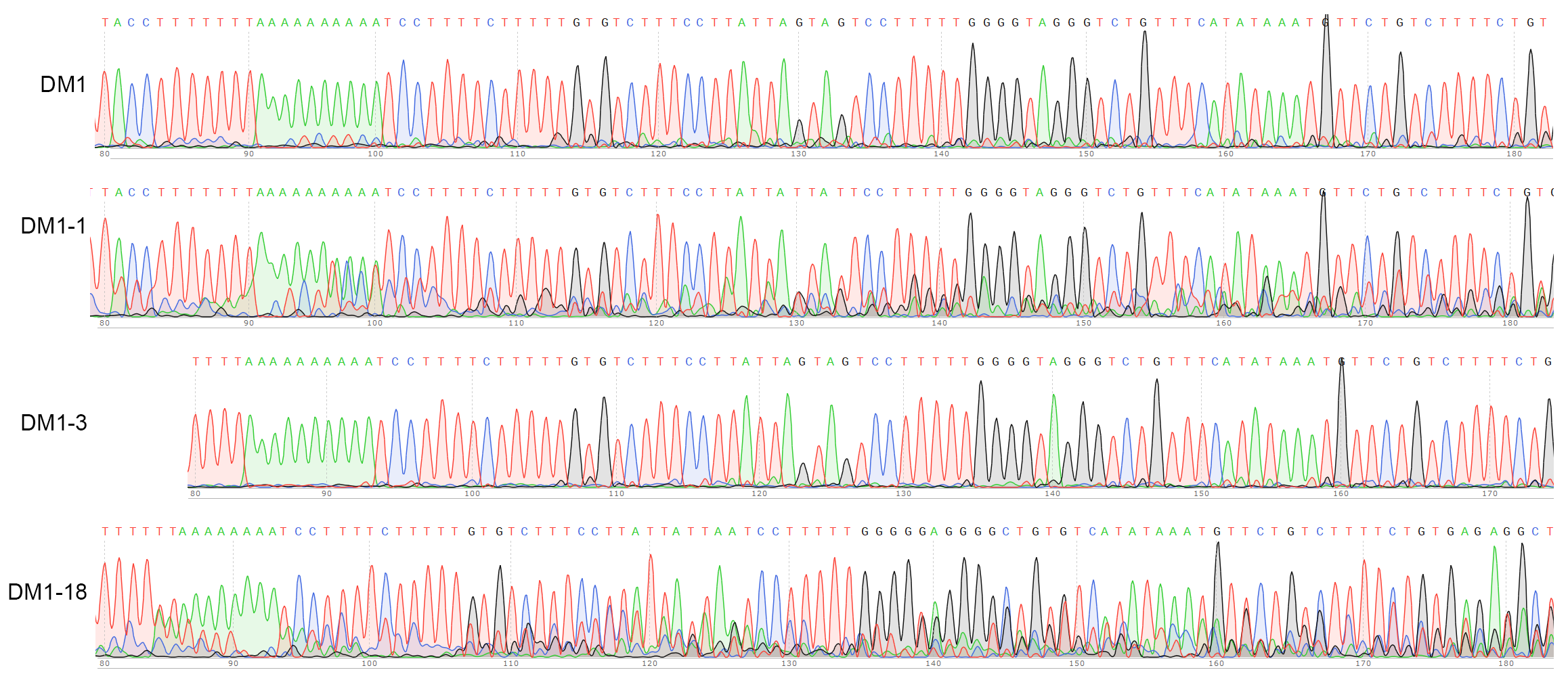

Supplement: Supplementary file 1 [file plants-15-00096-s001.zip › FigureS2m.tif]

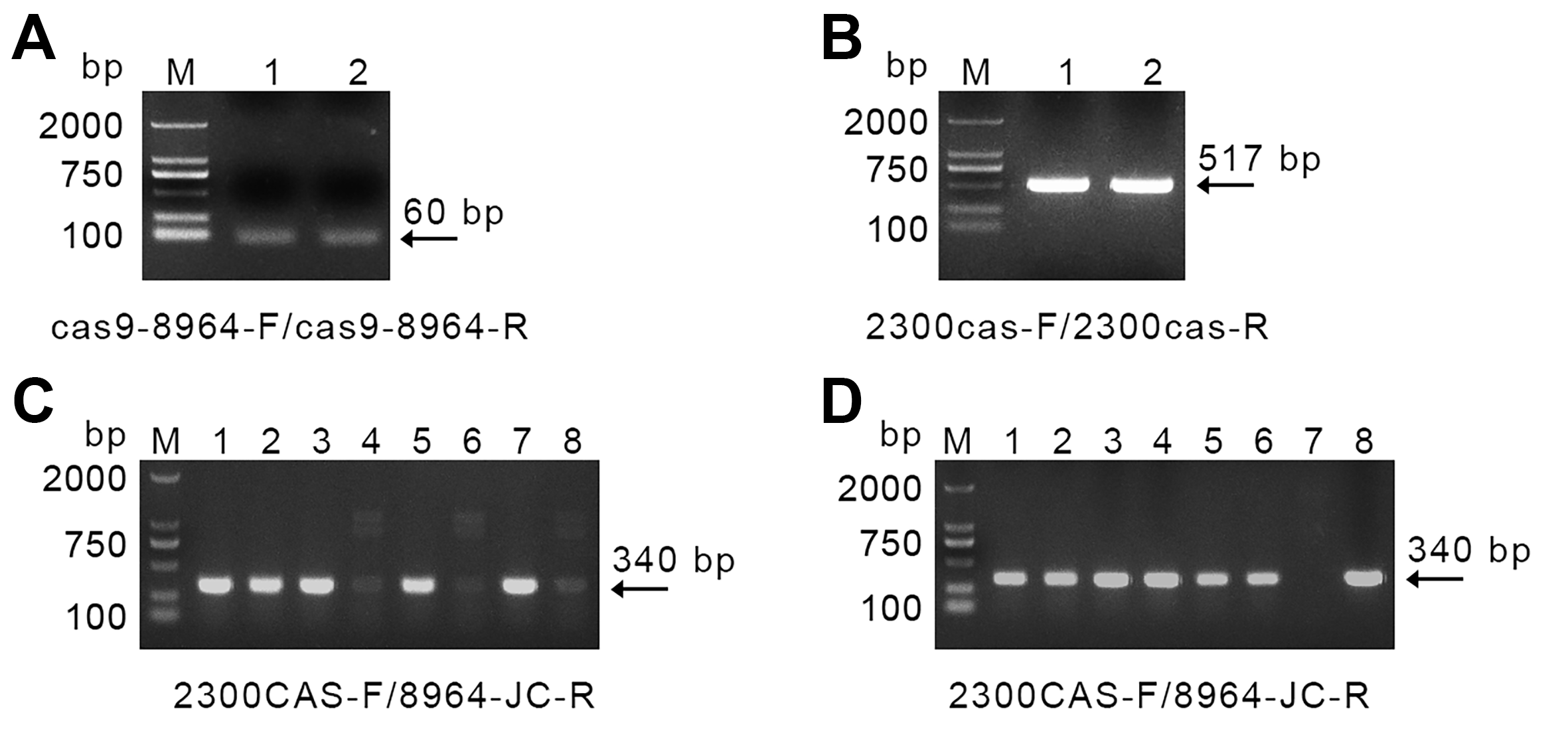

Supplement: Supplementary file 1 [file plants-15-00096-s001.zip › FigureS1m.tif]
